# Navigating reconfiguration: case managers in public municipal healthcare – a scoping review protocol

**DOI:** 10.1136/bmjopen-2026-124989

**Published:** 2026-10-20

**Authors:** Tove Kristin Hafsmo Yndestad, Martin S Krane, Cathrine Arntzen, Trude Anita Hartviksen

**Affiliations:** 1 Center for Care Sciences, North, UiT The Arctic University of Norway Faculty of Health Sciences, Tromsø, Troms, Norway; 2 Vestvågøy Municipality, Leknes, Norway

**Keywords:** Health policy, PUBLIC HEALTH, HEALTH ECONOMICS, STATISTICS & RESEARCH METHODS, Organisation of health services

## Abstract

**Abstract:**

**Introduction:**

Public municipal healthcare plays a critical role in delivering equitable, need-based services to the inhabitants. The term is used here for publicly financed services holding territory-based responsibility for the health of a defined resident population at municipal or comparable sub-national level. However, demographic shifts, multimorbidity and resource constraints have reinforced a need for systemic reconfiguration. Within the context of these organisational changes, the scoping review will address the current knowledge gap in understanding the roles, experiences and systemic impact of case managers and other caseworkers as key frontline professionals. The scoping review will consider primary research, publications by relevant and credible national and international websites of health organisations and agencies and public administration and governance bodies. The review aims to map existing literature on how case managers and other caseworkers navigate reconfiguration including organisational change, new models of service delivery, decentralisation and digitalisation.

**Methods and analysis:**

The JBI (formerly the Joanna Briggs Institute) methodology for scoping reviews and the Preferred Reporting Items for Systematic Reviews and Meta-Analysis extension for Scoping Reviews guidelines will be followed. The systematic search will include Medline (via PubMed), Web of Science, Cumulative Index to Nursing and Allied Health Literature (CINAHL), PsycINFO and Embase databases. Grey literature will be searched using Google Scholar and ProQuest. Searches will be conducted in September–December 2026. Screening of titles, abstracts and full-text articles will be independently conducted by two reviewers. Extracted data will be charted in tables and synthesised thematically. No geographic limits will be applied.

Using the Population-Concept-Context framework, inclusion criteria are (1) case managers and other caseworkers, (2) navigating reconfiguration of healthcare and (3) public municipal healthcare and equivalent sub-national arrangements. Exclusion criteria are (1) managers, health personnel, policymakers or clients without direct attention to case managers’ or caseworkers’ work, roles or experiences; (2) studies focusing on individual training programmes or clinical interventions that mention ‘navigation’ or ‘navigate’ only in passing, without substantively analysing reconfiguration of public municipal healthcare; and (3) studies set exclusively in hospitals, at the national policy level, or in settings outside municipal or community healthcare, or in services that are neither publicly financed nor publicly mandated.

**Ethics and dissemination:**

The scoping review will not require ethics approval, as all data will be obtained from publicly available literature. The review authors will adhere to principles of honesty and transparency throughout the data extraction and reporting processes and thereby enhance the trustworthiness of the review. Findings will be submitted for publication in a peer-reviewed journal and disseminated through presentations at relevant conferences.

**STRENGTHS AND LIMITATIONS OF THIS STUDY**

- This scoping review follows established JBI methodology and an a priori protocol registered on Open Science Framework (10.17605/OSF.IO/SD3TB), with reporting guided by Preferred Reporting Items for Systematic Reviews and Meta-Analysis extension for Scoping Reviews (PRISMA-ScR). Titles, abstracts and full texts will be screened independently by two reviewers.
- The search strategy was developed with a university librarian with expertise in literature searching and spans multiple databases with grey literature included to capture policy and municipal-sector sources that are typically absent from bibliographic indexes.
- Eligibility rests on territory-based responsibility for a defined resident population rather than on administrative labels, allowing the review to include comparable arrangements across differing national systems; governance context will be charted so that variation between systems is presented as a finding.
- A potential limitation is that inconsistent terminology for the case-manager role across countries, languages and disciplines means relevant records may be missed. The search strategy incorporates the range of titles used internationally, and terminological variation will be charted as a finding.
- Language restrictions may limit coverage of literature published, for example, in French, Portuguese and Japanese, including from systems named as relevant comparators. As is standard for scoping reviews, methodological quality of included sources will not be formally appraised.

## Introduction

 Primary healthcare (PHC) brings integrated health and care services closer to communities, spanning health promotion, disease prevention, treatment, rehabilitation and palliative care across the life course.^1^ PHC describes a level of care but does not determine who is responsible for providing it. In many systems that responsibility is territorial, a publicly financed, publicly accountable body holds a territory-based responsibility for population health, answerable for services to all inhabitants of a defined area. Municipal responsibility of this kind is a defining feature of the Nordic welfare states but not unique to them. Quebec, Brazil and Japan are examples of countries that also organise first-line services around comparable territorial mandates.^2–4^

Throughout the review, services organised in this way will be referred to as public municipal healthcare. The term denotes the governance arrangement rather than a particular administrative tier and includes district, communal and other locally governed equivalents. Where responsibility extends to a whole resident population, services are allocated by assessed need and legal entitlement rather than demand or ability to pay. This places equity and distributive justice at the core of their mandate.^5^ The review will include case managers and caseworkers whose organisations hold such a territory-based responsibility in accordance with this mandate and how they navigate its reconfiguration.

The equity of case managers and caseworkers’ mandate has become increasingly difficult to fulfil for several combined reasons. Populations worldwide are ageing rapidly due to declining birth rates and rising life expectancy, a demographic transition that significantly affects the social, economic and political conditions within which healthcare operates.^6^ Ageing also changes the composition of need. Multimorbidity, defined by WHO as the coexistence of two or more chronic health conditions in the same person,^7^ affects more than one in three adults globally and over half of those aged 60 and above.^8^ It generates complex needs across health, functional and social domains that require coordination rather than episodic treatment. Capacity is meanwhile constrained. WHO projects a global shortfall of more than 11 million health workers by 2030,^9^ with shortages falling hardest on rural and remote areas.^10^ Across Organisation for Economic Co-operation and Development countries, care is at the same time shifting out of hospitals towards outpatient, community and home-based settings.^11^ Where responsibility for such services is territorial, it follows that this shift places increasingly complex work in local hands.^4^

Strengthening PHC has been the dominant international response to such issues. The 2018 Declaration of Astana marked 40 years since Alma-Ata and was unanimously endorsed by all WHO Member States. It reaffirmed PHC as the foundation for universal health coverage and committed signatories to political choices across sectors, sustainable PHC, empowered communities and aligned stakeholder support.^12^ For services organised around territorial responsibility, honouring that commitment under these pressures requires reconfiguration. Here, reconfiguration is to be understood as deliberate, system-level changes in how care is structured, organised, financed and delivered, as opposed to incremental improvements within existing models. The term is used in this sense in research on hospital service reconfiguration in the English National Health Service, where the object of the analysis is the withdrawal or relocation of services rather than their internal improvement.^13^ No corresponding conceptualisation was found in municipal or first-line services, so the definition is extended here from the hospital literature.

Reconfiguration necessarily involves organisational change. It has been characterised as difficult, contentious and conflictual with implementation depending on conditions at both local and central levels and on the coordination between them.^13^ The centralisation of acute stroke services in London and Greater Manchester, both hospital based, illustrates this: the same intervention was implemented more fully in London, producing significantly different implementation and patient outcomes, including mortality.^14 15^ Differences in implementation have been found to track the content of the proposed changes and local political conditions rather than the strength of the evidence for change.^13^ A systematic review of whole-organisation transformation similarly found distributed leadership, staff engagement and culture change to be recurring themes in successful transformation, while noting that this literature remains methodologically weak.^16^ In Lipsky’s account of street-level bureaucracy, the decisions and routines of frontline staff effectively become the policy that is delivered.^17^ Reconfiguration is no different: what it becomes in practice depends on how frontline staff interpret and carry it out. The studies cited here, however, concern hospital services almost exclusively, while reconfiguration in territorially responsible first-line services and the role of case managers and case workers within them remain far less described.

Reconfiguration bears directly on the work of staff whose core function is to assess individual needs, determine eligibility for services, issue formal decisions on entitlement and coordinate or allocate publicly funded provision. When the range of available service changes, or the thresholds for qualifying for them are redrawn, these staff are the point at which reform becomes a decision about a particular resident.^17^ This population is referred to throughout as case managers and other caseworkers, recognising that the same function is performed under a wide range of titles and institutional arrangements, including care coordinators, service assessors, social caseworkers and navigators. An umbrella review of these roles found their tasks and responsibilities to be highly variable and often poorly defined.^18^

The significance of case managers and caseworkers extends beyond the individual services they facilitate. Their aggregated decisions constitute the primary allocation mechanism for scarce public resources in public municipal healthcare, with direct implications for both economic sustainability and workforce demand. They occupy positions that welfare state research identifies as street-level bureaucracy, where discretionary decisions become, in practice, the operative expression of public policy.^17^ Developed from studies of public agencies in the USA, including welfare offices, schools and police, this account has since been applied widely across welfare services. A systematic review of 165 studies of street-level coping found organisational conditions to be the strongest influence on frontline behaviour.^19^ Its application to need assessment and service allocation in territorially responsible first-line care is more recent and considerably less developed, although a growing literature examines how digital systems reshape frontline discretion.^20^

Despite this importance, the evidence concerning these professionals is fragmented. Existing literature approaches them from a small number of separate vantage points: as participants in case management intervention programmes, where a systematic review of 22 studies across six countries identified implementation barriers including physician understanding of the role, case manager autonomy and workload^21^ and as street-level bureaucrats in employment or social welfare services.^17 19^ Where they have been considered in relation to organisational change, attention to their role as active agents has been limited. A bibliometric review found individual acceptance of change in healthcare to be undeveloped as a field of study and only beginning to move beyond a framing of frontline staff as either change agents or resisters.^22^

A scoping and mapping review of case management found substantial variation in how the role is defined and modelled, which constrains synthesis across countries and settings.^23^ To our knowledge, no review has mapped the experiences, roles, working conditions and systemic effects of case managers and caseworkers navigating reconfiguration in public municipal healthcare. This review will therefore map and descriptively synthesise the available evidence on case managers and other caseworkers performing these functions to identify key concepts, evidence gaps and research trends, with particular attention to their roles, experiences and functions in the context of reconfiguration.

The relevant literature is dispersed across health service research, social work, public administration and gerontology. The role carries inconsistent titles across systems, and no settled definition exists. These are the conditions under which JBI guidance indicates a scoping review.^24 25^ The review will draw on empirical studies of any design together with grey literature. Because territorially responsible first-line care is realised differently across systems, the charting form will record jurisdiction, form of decentralisation and the breadth of local decision-making authority. This allows variation across contexts to be presented as a finding rather than treated as noise. The review constitutes the first stage of a doctoral project examining caseworkers in rural Norwegian municipalities. Mapping the international evidence establishes the comparative context against which that empirical work will be interpreted.

## Review question

The scoping review aims to map the existing literature on how case managers and other caseworkers in public municipal healthcare navigate reconfiguration, including organisational change, new models of service delivery, decentralisation and digitalisation. This aim is addressed through three sub-questions:

- What types of reconfigurations have been examined in relation to their work—including organisational reform, new models of service delivery, decentralisation or centralisation of responsibility, digitalisation of assessment and decision-making and change affected by demographic and workforce pressures?
- How do case managers and other caseworkers perceive, interpret, adapt to or make sense of reconfiguration in their daily work?
- How does reconfiguration alter their roles, responsibilities and practices—specifically their task content, scope of discretion, decision-making authority, workload, professional boundaries and relationships with service users, colleagues and management?

Sub-question two addresses the experiential dimension and will draw on qualitative studies and the qualitative components of mixed-methods studies, while sub-question three concerns documented change in the work itself and will draw on empirical studies of any design, together with policy and organisational documents reporting such change.

## Inclusion criteria

### Participants

The scoping review will consider studies that describe case managers and other caseworkers involved in assessing needs, determining eligibility, issuing formal decisions on service entitlement and allocating or coordinating public municipal healthcare as defined in the Introduction section. Case managers and other caseworkers may also include care managers or care coordinators, patient navigators or municipal nurses in case management roles.

Studies focusing only on managers, health personnel, policymakers or clients without direct attention to case managers’ or caseworkers’ work, roles or experiences will be excluded. Studies focusing solely on the delivery or provision of direct care services, without addressing the assessment, decision-making or coordination functions of case managers or other caseworkers, will also be excluded.

### Concept

The review will consider studies that examine how case managers and other caseworkers navigate, adapt to or make sense of the reconfiguration of healthcare, including their practices and perceptions in response to such changes. The reconfiguration of healthcare encompasses organisational changes, reforms, new models of service delivery, decentralisation or centralisation of responsibility and the digitalisation of assessment and decision-making, whether affected by demographic, workforce or fiscal pressures.

Studies focusing on individual training programmes or clinical interventions that mention ‘navigation’ or ‘navigate’ only in passing, without substantively analysing reconfiguration of public municipal healthcare, will be excluded.

### Context

No geographic limits will be applied. The review will consider studies conducted in publicly financed, publicly accountable services holding territory-based responsibility for the health of a defined resident population, including home care, rehabilitation, nursing homes, primary care and other community-based care services. Such services are variously organised at municipal, communal, district, county or comparable sub-national level. All of them will be eligible.

Studies set exclusively in hospitals, at the national policy level or in settings outside municipal or community healthcare, such as schools, prisons, will be excluded, as will studies of services that are neither publicly financed nor publicly mandated. Such studies may be included where municipal or community-based care constitutes a substantive part of the empirical setting and findings pertaining to that setting can be distinguished from those of other contexts.

To ensure consistent application of these criteria across systems, screeners will apply the following rule. A study is eligible where all four conditions are met: (1) the services concerned are the responsibility of a publicly accountable body at sub-national level; (2) that body holds responsibility for a defined resident population or territory, rather than only for its own enrolled or paying users; (3) the services are financed predominantly through government or compulsory contributory schemes, irrespective of whether delivery is public, contracted or mixed; and (4) the study reports on staff performing needs assessment, eligibility determination, formal decision-making on entitlement or service coordination and allocation within that body. The rule will be piloted as described under the Study selection section.

### Types of sources

Eligible sources will include both academic and grey literature. This includes primary research employing qualitative, quantitative or mixed-methods designs, publications by relevant and credible national and international websites of health organisations and agencies and public administration and governance bodies (eg, government websites on the management and allocation of municipal healthcare services).

To cast a wide net across source types, all sources will be considered, including peer-reviewed journal articles, book chapters, PhD dissertations and master’s theses, conference abstracts, reports and text and opinion papers.^24 25^ Text and opinion papers will be eligible only when the case managers or caseworkers are their principal subject.

Only studies published in English or Scandinavian languages will be included because of the language capabilities of the research team.

## Methods and analysis

The review will be conducted in accordance with the JBI methodology for scoping reviews,^26^ in addition to the foundational framework of Arksey and O’Malley.^25^ The Preferred Reporting Items for Systematic Reviews and Meta-Analyses Extension for Scoping Reviews (PRISMA-ScR)^27^ will be adhered to prepare the full report. This protocol was registered with the Open Science Framework (10.17605/OSF.IO/SD3TB).

### Search strategy

The search strategy will aim to locate both published and unpublished studies. A three-step search strategy will be used, in line with the JBI recommendations. First, an initial limited search of MEDLINE (via PubMed) and CINAHL (EBSCOhost) will be conducted to identify the initial keywords from the titles and abstracts of relevant articles, as well as the index terms used to describe them. Second, a comprehensive search will be performed using all the identified keywords and index terms across multiple databases, including PubMed, CINAHL (EBSCOhost), Web of Science, PsycINFO and Embase. These databases are selected to ensure broad and interdisciplinary coverage of relevant literature, spanning clinical medicine and public health, nursing and allied health, psychology and behavioural sciences and cross-disciplinary research across the natural and social sciences. For grey literature, a search in Google Scholar and ProQuest will be conducted. The comprehensive search will be conducted in September–December 2026. The search will be updated prior to submission of the completed review should more than 12 months elapse.

The search strategy will be adapted for each database (see online supplemental appendix 1 for the search strategy for CINAHL). Finally, the reference lists of all sources selected for full-text screening will be manually searched (backward citation searching) to identify additional relevant studies. Additionally, a forward citation search of the key articles will be conducted.

This review will include the literature published from 2010 onwards. Three developments justify this cut-off. First, the WHO World Health Report 2008 marked a renewal of PHC policy.^28^ Second, the 2008 global financial crisis introduced lasting fiscal pressure on healthcare.^29^ Third, integrated care frameworks became mainstream in both research and policy around this time.^30^ Together, these shifts created contextual conditions that were distinct enough that earlier literature is of limited relevance to the review’s aim.

### Study selection

Following the search, all identified citations will be collated and uploaded to Rayyan, and duplicates will be removed. The selection process will be piloted by two independent reviewers on a sample of approximately 25 citations to ensure the dependability of the eligibility criteria. Following the pilot test, titles and abstracts will be screened by two independent reviewers to assess the inclusion criteria for the review. Potentially relevant sources will be retrieved in full and imported into Rayyan to facilitate the management of the full-text review. Sources for which the full text cannot be retrieved, even after attempts to contact the authors, will be excluded at this stage. The full texts of the remaining sources will be assessed in detail against the inclusion criteria by two independent reviewers. Any disagreements between the reviewers at each stage of the selection process will be resolved through discussion or, if required, with an additional reviewer. The results of the search and study inclusion process will be reported in full in the final scoping review and presented in a Preferred Reporting Items for Systematic Reviews and Meta-Analyses (PRISMA) flow diagram.^31^

### Data extraction

Data will be extracted from papers included in the scoping review by two independent reviewers, using a data extraction tool developed by the reviewers. The extracted data will include specific details about the participants, concept, context, study methods and key findings relevant to the review question.

Online supplemental appendix 2 provides the draft extraction form (see online supplemental appendix 2). The draft data extraction tool will be modified and revised as necessary during the process of extracting data from each of the included evidence sources. The modifications will be detailed in the scoping review. Any disagreements between the reviewers will be resolved through discussion or by an additional reviewer. If appropriate, the authors of the papers will be contacted to request missing or additional data, where required.

### Data analysis and presentation

To analyse and synthesise the data, a descriptive synthesis will be conducted, consistent with the JBI scoping review methodology. Data will be extracted using a structured form developed in Excel, and coding of extracted findings will be managed in NVivo. The extracted data will first be charted to summarise source characteristics, the governance context and jurisdiction of the services studied, the population served, how the case manager role is defined and what tasks and responsibilities it carries, the type of reconfiguration examined and the conditions under which it was implemented and reported outcomes relevant to the review questions. Because territorially responsible first-line care is organised differently across systems, the charting form records the governance arrangement of each included setting, allowing variation across contexts to be presented as findings of the review. While charting provides the descriptive map, the second sub-question concerns perception and sense-making, and the relevant findings will be reported in the analytic language of each included study. A thematic synthesis, informed by the reflexive thematic analysis framework of Braun and Clarke,^32^ will therefore be applied to pattern these findings across differing terminologies and settings, making the interpretive work explicit rather than implicit.

### Patient and public involvement

Patients or public were not involved in this part of the research.

## Ethics and dissemination

The scoping review does not require ethical approval, as it will exclusively use data from publicly available and previously published literature. All steps in the review process will be documented and reported transparently, in line with PRISMA-ScR, to support reproducibility and the trustworthiness of the findings.^33^

The review will be conducted by two reviewers. Screening and full-text assessment will be carried out independently by two reviewers at each stage, with inter-rater agreement reported for the pilot; disagreements will be resolved by discussion or by a third reviewer. Data extraction will be undertaken independently by two reviewers.

The review will produce a map of the types of reconfigurations examined in relation to case managers and other caseworkers of how these professionals experience and respond to such change and of how their roles and practices are reported to alter. Charting the governance context of each included setting will allow this map to be read across differing national arrangements rather than within a single one. The review is intended to serve several audiences; researchers, for whom it identifies where evidence is concentrated and where it is absent; managers and policymakers in territorially responsible services, for whom it assembles what is known about a professional group central to allocation decisions; the case managers and caseworkers themselves and the wider doctoral project, for which it establishes the comparative context against which empirical work in Norwegian municipalities will be interpreted. Constraints on the review are set out in the Strengths and limitations section.

The presentation of the results will be structured around the review questions. The review will be submitted for publication in an international peer-reviewed journal, and the results will be presented to stakeholders and, if possible, in appropriate national and international conferences.

## Supplementary material

10.1136/bmjopen-2026-124989 online supplemental appendix 1

10.1136/bmjopen-2026-124989 online supplemental appendix 2
