# Supplementary material for: Navigating reconfiguration: case managers in public municipal healthcare – a scoping review protocol
Source: BMJ Open. 2026 Oct 20;16(10):e124989. doi: 10.1136/bmjopen-2026-124989 (PMC13684227; doi:10.1136/bmjopen-2026-124989)
Supplement: Supplementary file 1 [file bmjopen-16-10-s001.pdf]

Template for systematic and structured literature searches

	Main concept 1	Main concept 2	Main concept 3
**Main concept**	**Case manager**	**Reconfiguration of health services**	**Municipal health/ public health**
**Controlled vocabulary search term**<br>(MeSH-terms, CINAHL Subject Headings, Emtree-terms, etc)<br>*Remember to consider using 'Explode'*	S1 (MH "Case Management") OR (MH "Case Managers")	S4 (MH "Health Care Reform") OR (MH "Organizational Change") OR (MH "Health Services Administration") OR (MH "Health Care Delivery, Integrated") OR (MH "Strategic Planning") OR (MH "Health Facility Merger")	S7 (MH "Public Health") OR (MH "Public Health Nursing") OR (MH "Community Health Services") OR (MH "Community Health Nursing") OR (MH "Local Government") OR (MH "Primary Health Care")
**'Free' text words**<br>(Synonymous terms, British/American spelling, abbreviations, acronyms, etc.)<br>*Consider using: field codes (title, abstract, etc), phrase searches, truncation, wildcards, proximity operators*	S2 XB ("case manager*" OR "case management" OR "care coordinat*" OR "care manager*" OR "care navigat*")<br><br>S3: S1 OR S2	S5 XB (reconfigur* OR reorganis* OR reorganiz* OR "re-organiz*" OR restructur* OR "service development" OR transformation OR	S8 XB (municipal* OR "public health" OR "community health" OR "community care" OR "community-based" OR "primary care" OR "primary health care" OR "local

		"integrated care" OR "care integration" OR ((service* OR health* OR care) N2 reform*) OR (service* N2 (chang* OR redesign*)))<br><br>S6: S4 OR S5	government" OR "local authorit*")<br><br>S9: S7 OR S8
	Search result 1 (#1) =	Search result 2 (#2) =	Search result 3 (#3) =

Final combination: S10: S3 AND S6 AND S9

S10: (S1 OR S2) AND (S4 OR S5) AND (S7 OR S8): **1 169**

(MH "Case Management") OR (MH "Case Managers") OR XB ("case manager*" OR "case management" OR "care coordinat*" OR "care manager*" OR "care navigat*")

AND

(MH "Health Care Reform") OR (MH "Organizational Change") OR (MH "Health Services Administration") OR (MH "Health Care Delivery, Integrated") OR (MH "Strategic Planning") OR (MH "Health Facility Merger") OR XB (reconfigur* OR reorganis* OR reorganiz* OR "re-organiz*" OR restructur* OR "service development" OR transformation OR "integrated care" OR "care integration" OR ((service* OR health* OR care) N2 reform*) OR (service* N2 (chang* OR redesign*)))

AND

(MH "Public Health") OR (MH "Public Health Nursing") OR (MH "Community Health Services") OR (MH "Community Health Nursing") OR (MH "Local Government") OR (MH "Primary Health Care") OR XB (municipal* OR "public health" OR "community health" OR "community care" OR "community-based" OR "primary care" OR "primary health care" OR "local government" OR "local authorit*")
