# Supplementary material for: Navigating reconfiguration: case managers in public municipal healthcare – a scoping review protocol
Source: BMJ Open. 2026 Oct 20;16(10):e124989. doi: 10.1136/bmjopen-2026-124989 (PMC13684227; doi:10.1136/bmjopen-2026-124989)
Supplement: Supplementary file 2 [file bmjopen-16-10-s002.pdf]

Extraction form, scoping review

Author, year, country	Aim	Participants	Method, Data analysis	Context	Type of reconfiguration	Result
