# Supplementary material for: Navigating reconfiguration: case managers in public municipal healthcare – a scoping review protocol
Source: BMJ Open. 2026 Oct 20;16(10):e124989. doi: 10.1136/bmjopen-2026-124989 (PMC13684227; doi:10.1136/bmjopen-2026-124989)
Supplement: Supplementary file 3 [file bmjopen-2026-124989.reviewer_comments.pdf]

PEER REVIEW HISTORY

BMJ Open publishes all reviews undertaken for accepted manuscripts. Reviewers are asked to complete a checklist review form and are provided with free text boxes to elaborate on their assessment. These free text comments are reproduced below.

ARTICLE DETAILS

Title (Provisional)

Navigating Reconfiguration: Case Managers in Public Municipal Healthcare - A Scoping Review Protocol

Authors

Yndestad, Tove Kristin Hafsmo; Krane, Martin S.; Arntzen , Cathrine; Hartviksen, Trude Anita

---

VERSION 1 - REVIEW

**Reviewer**	**1**
**Name**	**Dionne, Emilie**
**Affiliation**	**Universite Laval**
**Date**	**17-Aug-2026**
**COI**	**None**

This is a protocol paper presenting a study to conduct a scoping review on the topic of case managers operating in the context of public municipal healthcare, in the face of healthcare reconfigurations (reforms, reorganizations). This topic is of great relevance, as case managers are emerging as particularly needed and in-demand professionals in the context of increasing and more complex healthcare needs and issues, entangling the social and health dimensions. I therefore consider it an important topic to study.

The protocol also aims to shed light/consolidate knowledge in a context where reforms/ reconfigurations of primary healthcare worldwide have been done by embracing principles of universal care, equity, and justice, whereby health organizations were endowed with the responsibility to ensure population health on a given, geographic area. Municipalities, in this context, were therefore “enrolled”, and notably as the concept of “health” itself has been revised, renewed, improved, now integrating determinants of health, prevention, promotion, health habits, access to services across the spectrum of human needs and activities – social, educational, cultural, natural/environmental, employment, security, and so forth.

In terms of my scientific assessment: Overall this protocol paper has much merit, yet in its current form, there are important issues/elements that need to be addressed, resolved,

clarified. It is therefore not currently ready for publication, but it can be by providing clarifications for these noted elements (i.e. in my opinion, issues are minor; can all be resolved rapidly). Mainly, clarifications are needed (this pertains to the engagement with the literature, directly in the text; and references provided — at times, these are lacking); sentence structures that must be revised, syntax is wrong, wording; some important details about the study and its aims are missing (concepts, analyses; applications). The most important revisions concern conceptual clarification, contextualization, the articulation of the literature gap, and the level of detail provided about the planned analysis.

The main issue relates to the concepts used for this study. There are many, and most require operational definitions not only for the study itself, but also to support a fair evaluation of the protocol. Specifically, primary healthcare is eventually defined by the authors, but I still think the definition used needs to be presented more directly and clearly. Second, the authors are specifically interested in public municipal healthcare. I am based in North America, and this is a novel concept or way of presenting this professional type, one I am not accustomed with, but find quite interesting and important. Yet in reading the protocol, it is not quite clear how the authors propose to operationalize this concept… or its idea. I highly recommend that the authors provide a clear and precise definition of this concept, perhaps with some historical background and contextualization; and clarify 'how/where' they will look, when selecting studies. I don't think, in other words, that this term is ubiquitously used. This clarification seems important because the concept directly affects the search strategy, inclusion and exclusion criteria, and interpretation/reach of the findings. The authors are likely interested in primary healthcare case managers whose organizations now abide by a territory-based responsibility for population health. It is not clear to me that the way the term is currently presented, notably for the actual review, will capture what they seek.

Then, contextualization, in my opinion, will yield much of the value and richness of the data that will be found and analysed. Yet I find that the authors say too little about context: how it may be relevant to them, and how they plan to consider it in their data selection and analyses. My annotated version clarifies all the places where I think engagement with "contexts", being more precise, providing concrete examples, too, will be helpful – for your study, for your audience.

This was notably true in the context of the literature presented. In the current version of the manuscript, I found it difficult to grasp the gap clearly. Studies are referenced, but they are not adequately presented or described in the text itself. We have to look at the references to get some information about them. However, this should be discussed and shown, clearly and concretely, in the text; and contexts, discussed, provided — What are the texts and studies that you cite, and why? Where does the data you are discussing come from, and from which regions or contexts? And so forth. The annotated copy of the protocol provides suggestions; this does not need to be extensive. If you know the texts, you will most often be able to add a few words within the sentences to meet this informational need — but there are places

where I think you should cite studies. Affirmations are made without appropriate referencing.

Now, the section on “analyses.” I find it too short and lacking important details. Please expand further on how descriptive data will be extracted and which descriptive data will be extracted. For example, the authors could clarify whether they will extract information on context, jurisdiction, organizational model, role definition, population served, tasks and responsibilities, outcomes, and implementation conditions. I suspect they will find not only a considerable amount of information and data, but also that the usefulness of the review will depend on how this information is analysed, made sense of, organized, compared, and contrasted.

Finally, consider adding two sections: (1) the contribution to knowledge and decision-making processes, including how the outcomes of this study are envisioned to help and whom they may help, such as researchers, organizations, professionals, case managers themselves, municipal governments, and other decision- or policy-making levels; international bodies, too; and (2) the potential limitations and limits of your study. These additions would help readers better understand the expected value, scope, and boundaries of the proposed review.

I hope these comments and suggestions will be helpful. I am available to assess an updated version of the protocol.

**** **The reviewer provided a marked copy with additional comments. Please contact the publisher for full details**. ****

VERSION 1 - AUTHOR RESPONSE

Reviewer 1

Part A – Response to the main review

1. Mainly, clarifications are needed (this pertains to the engagement with the literature, directly in the text; and references provided — at times, these are lacking); sentence structures that must be revised, syntax is wrong, wording; some important details about the study and its aims are missing (concepts, analyses; applications).

We are grateful for this comment and have revised the manuscript on all four counts.

2. The most important revisions concern conceptual clarification, contextualization, the articulation of the literature gap, and the level of detail provided about the planned analysis.

We have revised the manuscript on all four counts. Each point is addressed in the responses below, at the annotations where it arises, together with the sentence-level corrections.

3. The main issue relates to the concepts used for this study. There are many, and most require operational definitions not only for the study itself, but also to support a fair evaluation of the protocol. Specifically, primary healthcare is eventually defined by the authors, but I still think the definition used needs to be presented more directly and clearly.

We have now defined Primary health care in the opening two sentences of the Introduction (page 3, lines 75–78), before any other concept is introduced, and the definition distinguishes the level of care from the question of who is responsible for providing it. Multimorbidity, previously used without definition, is now defined following WHO (page 3, lines 96–97), with a note that the term is itself variously defined in the literature and a statement of which definition governs our use. Reconfiguration is defined at page 3, lines 112–114.

4. Second, the authors are specifically interested in public municipal healthcare. I am based in North America, and this is a novel concept or way of presenting this professional type, one I am not accustomed with, but find quite interesting and important. Yet in reading the protocol, it is not quite clear how the authors propose to operationalize this concept… or its idea. I highly recommend that the authors provide a clear and precise definition of this concept, perhaps with some historical background and contextualization; and clarify 'how/where' they will look, when selecting studies. I don't think, in other words, that this term is ubiquitously used.

We agree that the term is not ubiquitously used, and this observation prompted on of the most substantial revision to the manuscript. The concept is now defined by governance arrangement rather than by administrative label, with non-Nordic instances named — including Quebec's responsabilité populationnelle, Brazilian municipalização under the SUS, and Japanese municipal administration of long-term care insurance (page 3, lines 78–83). How and where we will look is addressed through a rewritten Context section and an explicit four-condition screening rule (pages 6–7, lines 227–247). Both are set out in full under annotations 1 and 11 below.

5. This clarification seems important because the concept directly affects the search strategy, inclusion and exclusion criteria, and interpretation/reach of the findings. The authors are likely interested in primary healthcare case managers whose organizations now abide by a territory-based responsibility for population health. It is not clear to me that the way the term is currently presented, notably for the actual review, will capture what they seek.

We are grateful for this formulation, which we have adopted as the defining criterion of the review. It now governs the definition in the Introduction (page 3, lines 78–80), the Context eligibility criterion (page 6, lines 231–236), the screening rule (page 7, lines 243–251), and the charting of governance context (page 9, lines 323–333). The search strategy has been revised accordingly, and the comprehensive search has not yet been run, so the revised strategy will be applied from the outset rather than retrofitted.

6. Then, contextualization, in my opinion, will yield much of the value and richness of the data that will be found and analysed. Yet I find that the authors say too little about context: how it may be relevant to them, and how they plan to consider it in their data selection and analyses. My annotated version clarifies all the places where I think engagement with "contexts", being

more precise, providing concrete examples, too, will be helpful – for your study, for your audience.

This was a really valuable comment, and context is now treated as a substantive element of the review rather than as background. The Introduction names concrete national instances (page 3, lines 80–83) and gives a worked example of how local and central coordination shapes reconfiguration outcomes, drawing on the centralisation of acute stroke services in London and Greater Manchester (page 4, lines 121-124). In data selection, context enters through the Context criterion and screening rule. In analysis, the charting form now records jurisdiction, the organisational model of the responsible body, the form of decentralisation and the breadth of local decision-making authority, so that variation across systems is presented as a finding of the review rather than treated as unexplained heterogeneity.

7. This was notably true in the context of the literature presented. In the current version of the manuscript, I found it difficult to grasp the gap clearly. Studies are referenced, but they are not adequately presented or described in the text itself. We have to look at the references to get some information about them. However, this should be discussed and shown, clearly and concretely, in the text; and contexts, discussed, provided — What are the texts and studies that you cite, and why? Where does the data you are discussing come from, and from which regions or contexts? And so forth. The annotated copy of the protocol provides suggestions; this does not need to be extensive. If you know the texts, you will most often be able to add a few words within the sentences to meet this informational need — but there are places where I think you should cite studies. Affirmations are made without appropriate referencing.

Thank you for this comment. Throughout the Introduction, cited studies are now described in the text rather than left to the reference list. Where a claim rests on a specific source, we state what kind of study it was, where its evidence comes from, and what it found — for example, the number of studies and countries covered by each systematic review cited, the settings from which the street-level bureaucracy account derives, and the English NHS regions compared in the stroke centralisation studies.

In doing so several references that did not support the claims to which they were attached were identified. These have been replaced or reattributed, and the claims adjusted to match what the sources establish. The gap statement no longer rests on citations to an absence; it now follows from what the preceding sentences demonstrate about what existing reviews have and have not covered (page 4, lines 132-135).

8. Now, the section on “analyses.” I find it too short and lacking important details. Please expand further on how descriptive data will be extracted and which descriptive data will be extracted. For example, the authors could clarify whether they will extract information on context, jurisdiction, organizational model, role definition, population served, tasks and responsibilities, outcomes, and implementation conditions. I suspect they will find not only a considerable amount of information and data, but also that the usefulness of the review will depend on how this information is analysed, made sense of, organized, compared, and contrasted.

This was a really valuable comment, and we have followed this suggestion directly. The charting form now records context and jurisdiction, the organisational model of the responsible body, how the case manager role is defined, the population served, tasks and responsibilities, the

conditions under which reconfiguration was implemented, and reported outcomes, alongside source characteristics, setting, reconfiguration type and study methods (page 9, lines 323–333). The detailed field list is set out in Appendix 2, where the form is structured by sub-question so that findings relevant to each are charted separately. The software to be used is now specified, and we have added a justification for combining descriptive synthesis with reflexive thematic analysis. Details are given under annotation 12.

9. Finally, consider adding two sections: (1) the contribution to knowledge and decision-making processes, including how the outcomes of this study are envisioned to help and whom they may help, such as researchers, organizations, professionals, case managers themselves, municipal governments, and other decision- or policy-making levels; international bodies, too; and (2) the potential limitations and limits of your study. These additions would help readers better understand the expected value, scope, and boundaries of the proposed review.

Thank you for these suggestions. We have added a paragraph on outcomes, contributions and applications to Ethics and dissemination (page 9, lines 338–358), describing what the review will produce and identifying the audiences it is intended to serve: researchers, managers and policymakers in territorially responsible services, case managers and caseworkers themselves, and the doctoral project of which the review forms the first stage.

For the second suggested section, we have expanded the existing Strengths and limitations of this study section (page 2, lines 53–72) rather than adding a separate one, since BMJ Open requires that section and a second treatment would have duplicated it. Details are given under annotation 13.

Part B – Responses to the annotations in the manuscript

1. Nordic framing of the concept (page 3, lines 78-91)

1.1 Given how the concept seems to emerge from the Nordic model, consider either expanding your concept/definition; or focusing on this region/context, for the scoping

Thank you for this comment, we agree that the concept as originally presented was tied too closely to the Nordic context, and that our own framing invited this reading.

Of the two options offered, we have chosen the first: to expand the concept and definition rather than to restrict the review geographically. First, JBI positions scoping reviews as the appropriate design for clarifying concepts and mapping heterogeneous evidence where no settled definition exists, which makes conceptual breadth methodologically indicated here rather than merely preferable. Second, on examination the phenomenon is not Nordic-specific: territorial public responsibility for first-line health and care services is a recurring international arrangement, found in Quebec's responsabilité populationnelle, Brazilian municipalização under the SUS, Chilean municipal primary care, Philippine devolution under the 1991 Local Government Code, and Japanese municipal administration of long-term care insurance.

The claim that municipal embedding is a defining feature of the Nordic model is retained but now qualified, with the Nordic arrangement appearing as one instance of a wider pattern, with non-Nordic examples named in the opening paragraph of Introduction (page 3, lines 78-91). We thank the reviewer for the suggestion offered and have adopted the formulation territory-based

responsibility for population health, as the defining criterion, which we agree captures the attribute more precisely than "municipal" did. We have retained "public municipal healthcare" as the recurring term, for continuity with the title and with the literature from which the review originates, but the Introduction now states explicitly that it denotes the governance arrangement rather than a particular administrative tier, and includes district, communal and other locally governed equivalents (page 3, lines 85-86).

The revised framing is intended to make that reach explicit rather than leaving it to inference.

2. Pressures on primary health care and the Declaration of Astana (page 3, lines 92-118)

2.1 Citations needed [lines 92-105]

We have now inverted the structure so that the pressures come first and the resulting difficulty follows as a conclusion, which addresses the request for a citation at that point. The claim is now supported by the evidence preceding it.

2.2 Is declining birth rate and rising life expectancy the sole challenge

We agree that demographic transition is not the sole challenge. The paragraph now identifies pressures on several fronts, each separately referenced. First, the changing composition of need multimorbidity is now defined, following WHO, as the coexistence of two or more chronic health conditions in the same individual, with prevalence drawn from a meta-analysis of 126 studies across 54 countries, and the resulting needs characterised as spanning health, functional and social domains. In response to the reviewer's principal comment on conceptual precision, we have noted that multimorbidity is itself variously defined in the literature and state which definition governs our use of the term. Second, constrained capacity, including WHO's revised projection of a shortfall of more than 11 million health workers by 2030 and its uneven distribution across rural and remote areas. Third, the relocation of care from hospitals towards outpatient, community and home-based settings.

2.3 Is [growing resource constraint] true everywhere?

Thank you for this comment, statements about budgetary constraint that we could not support across systems have been removed. The claim about care settings is now explicitly scoped to OECD countries.

2.4 This paragraph needs to be better referenced

Each substantive claim in the paragraph now carries its own reference, and the concluding sentence is marked as an inference and supported by the Japanese case (page 3, lines 103-105).

3. The Declaration of Astana and the definition of reconfiguration (pages 3-4, lines 106–118)

3.1 Elaborate, provide details

Thank you for this comment. The material duplicating the pressures has been removed. The account of the Declaration of Astana now specifies the Global Conference on Primary Health Care held in Astana on 25 and 26 October 2018, convened by WHO, UNICEF and the Government of Kazakhstan to mark the 40th anniversary of the Declaration of Alma-Ata, its unanimous endorsement by all WHO Member States, and its four commitment areas. The paragraph now closes on the definition of reconfiguration, which is now attributed to its use in Fulop and colleagues' study of hospital service on reconfiguration across three English NHS sites, rather than presented as a settled definition. The sentence on care increasingly provided in service users' homes has been removed, as that point is now made in the preceding paragraph with appropriate scoping and a reference.

4. Organisational change and reconfiguration (page 4, lines 119-135)

<u>**4.1** References are needed, perhaps concrete examples</u>

This was a really valuable comment and the characterisation of reconfiguration as difficult, contentious and conflictual is now referenced, supported by a systematic review of whole-organisation transformation that identifies distributed leadership, staff engagement and culture changing as recurring themes in successful change. We have also added a concrete example. Drawing on the comparison of acute stroke service centralisation in London and Greater Manchester, the paragraph shows that the same intervention was implemented more fully in London than in Manchester, with significantly different implementation and patient outcomes, including mortality. This provides empirical rather than asserted support for the claim that coordination between local and central levels shapes outcomes.

<u>**4.2** Revise [the enumeration of occupational groups]</u>

Thank you for pointing this out, we have now removed the enumeration of occupational groups, which was both imprecise and internally inconsistent. More substantially, we have reframed the account of frontline involvement. The previous version presented staff primarily as resisting change out of concern for their own position, which sat uneasily with the theoretical basis of the review, and the paragraph now reflects the understanding of frontline workers as exercising discretion that shapes what reform becomes in practice.

<u>**4.3** [The statement on human resource issues is] too vague; clarify and nuance</u>

Thank you for this comment. The vague statement about human resource issues has been replaced with a specific, referenced finding. Differences in implementation have been found to track the content of proposed changes and local political conditions rather than the strength of the evidence for change, while national policy has tended to over-emphasise evidence-based consultation and underplay these factors.

<u>**4.4** [The closing claim] seems trivially true</u>

Thank you for this comment. The paragraph now closes by identifying what this literature does not cover, drawing on the studies already cited rather than on a citation to an absence. The evidence on reconfiguration is concentrated almost entirely on hospital services, while reconfiguration processes in territorially responsible first-line services, and the position of case managers and case workers within them, remain less well described. This gap is the rationale for the present review.

5. Population, and terminology (page 4, lines 136-145)

5.1 Awkward phrasing; revise

Thank you for pointing this out, the opening sentence has now been rewritten to remove the awkward construction. We have also replaced the comparative claim that these professionals are "most exposed to and consequential" for reconfiguration, which we could not support, with an explanatory account: reconfiguration alters the range of available services and the threshold for qualifying for them, and these staff are the point at which such changes become decisions about particular residents.

The naming decision is now supported rather than asserted. An umbrella review of care navigator roles found tasks and responsibilities across these titles to be highly variable and often poorly defined, which is our reason for treating them as one population.

5.2 At this point, it would be essential to situate better, contextualise, your study… and literature review (page 5, lines 178-188)

This was a really valuable comment. Two changes have been made to improve the matter. We have added an explicit justification for the choice of design: the literature on these professionals is dispersed across health services research, social work, public administration and gerontology, the role carries inconsistent titles across systems, and no settled definition exists. These are the conditions under which JBI guidance indicates a scoping review rather than a systematic review. The passage now situates the review within our own programme of work, as the first stage of a doctoral project examining caseworkers in rural, Norwegian municipalities, with the international evidence establishing the comparative context against which that empirical work will be interpreted.

6. Significance and the street-level bureaucracy account (page 4, lines 149-157)

6.1 Contexts?

We have added a statement where this account originates – studies of public agencies in the United States, including welfare offices, schools, police and lower courts – and note that it has been applied widely across welfare services, particularly in employment and social services, while its application to needs assessment and service allocation in territorially responsible first-line care is more recent and considerably less developed.

7. Fragmentation and the gap (page 5, lines 158-167)

7.1 Syntax and structure to be revised

The original construction treated the professionals as "narrow lenses", which was a category error. The paragraph now presents the separate vantage points from which they have been studied. The unclosed parenthesis has been corrected.

7.2 The single citation – only 1 study cited… if the topic is understudied, indicate

Thank you for your valuable remark. Rather than adding citations we could not substantiate, we have followed the reviewer's suggestion and made the scarcity part of the argument. The paragraph now notes that few studies address this directly and that the scarcity itself indicates how little the topic has been studied. We have also softened the claim that there has been "no recognition" of these workers as active agents in change processes, since an absolute negative of that kind cannot be demonstrated, to state that such attention has been limited.

More broadly, each of the vantage points from which these professionals have been studied is now described in the text, with the scope and findings of the relevant review stated. The concluding claim that no review has mapped our topic follows from what the preceding sentences establish, rather than resting on citations to an absence.

7.3 Revise sentence structure

The concluding sentence has been restructured into the active voice.

8. Aim of the review (page 5, lines 173-176)

8.1 Revise sentence structure

In the aim paragraph, the original stated that a gap would be "mapped and synthesized", whereas what is mapped is the evidence, and the sentence now says so.

8.2 The introduction should say more about the contextualisation of your data and analyses

Thank you for this comment. The passage (page 5, lines 178-188) now describes what the review will draw on and how it will be handled: empirical studies of any design together with grey literature, charted using a structured extraction form and synthesised descriptively rather than statistically. Because territorially responsible first-line care is realised differently across systems, the charting form will record jurisdiction, form of decentralisation, and the breadth of local decision-making authority, allowing variation across contexts to be presented as a finding of the review.

9. Review question and sub-questions (pages 5-6, lines 189-207)

9.1 Consider expanding – digital turn?

Thank you for this comment. We have adopted the suggestion on digitalisation. On reflection, digitalisation is not simply one reconfiguration type among several, it acts directly on the functions that define our study population – needs assessment, eligibility determination, documentation and decision-making – and therefore on the discretion that makes these professional theoretically significant. We have added a brief statement to the Introduction noting the growing literature on how digital systems reshape frontline discretion (page 4, lines 156-157), citing Busch and Henriksen's systematic review of digital discretion. The first sub-question now names the digitalisation of assessment and decision making explicitly.

9.2 Please clarify the last two. The second one speaks to qualitative/social data – what types of study designs are you seeking for the third one?

This was a really valuable comment which identified a genuine ambiguity in the two sub-questions. As written, they could have been read as addressing the same material from two

directions, and we had not specified what evidence would answer the third. We have now distinguished them dimension and evidence base (lines 198-203). The second sub-question concerns the experiential dimension – how these professionals perceive, interpret, adapt to and make sense of reconfiguration – and will draw on qualitative studies and the qualitative components of mixed-methods studies, as the reviewer anticipated. The third sub-question concerns documented change in the work itself, and its object is now concrete: task content, scope of discretion, decision-making authority, workload, professional boundaries, and relationships with service users, colleagues and management. This question will draw on empirical studies of any design, including quantitative and mixed-methods studies, together with policy and organisational documents reporting such change.

We have additionally restructured the review question as a single overarching question with three sub-questions, rather than three parallel questions, reflecting standard JBI practice and making clearer that the three address facets of one inquiry. We have removed the qualifying clause referring to organizational change affected by demographic trends, which was inconsistent with the revised Introduction, and replaced it with the forms of reconfiguration the review will consider – organisational change, new models of service delivery, decentralisation and digitalisation – consistent with the abstract and the Concept eligibility criterion.

10. Eligibility criteria – Participants (page 6, lines 208-219)

<u>**10.1** Clarify [participants]</u>

Thank you for pointing this out, the exclusion criterion has now been revised in two respects. The qualifying phrase has been changed from "without substantive data on" to "without direct attention to" (page 6, lines 215-216), which states the test more clearly and does not depend on the presence of empirical data. The scope has been broadened from "experiences" to "work, roles or experiences" (line 216) so that studies reporting documented change in task content, discretion or workload remain eligible, consistent with the third sub-question.

The first of these changes also resolves an inconsistency with the Type of sources section (page 7, lines 252-263), that was found in revising it: the criterion previously required substantive data, while text and opinion papers are listed as eligible sources and by nature report none. Both sections now apply the same test, and Types of sources specifies that text and opinion papers are eligible where case managers and other caseworkers are their principal subject (page 7, lines 260-261).

11. Eligibility criteria – Context (pages 6-7, lines 230-251)

<u>**11.1** Worldwide?</u>

Thank you for pointing this out. The query identified an omission: the protocol did not state whether the review was geographically bounded, leaving this to be inferred from the term "municipal". The section now states explicitly that no geographic limits will be applied (line 231.)

More substantially, we have rewritten the section so that eligibility rests on the defining criterion rather than on an administrative label. Studies are eligible where services are publicly financed, publicly accountable, and hold territory-based responsibility for the health of a defined resident population at municipal, communal, district, county or comparable sub-national level. This

ensures that the arrangements named in the Introduction – in Quebec, Brazil and Japan among others – fall within scope, which the previous wording would not reliably have achieved.

We have also corrected an inconsistency: the original criteria excluded private-sector organisations, which conflicted with our own definition of public municipal healthcare as covering services that may be publicly, contractually or jointly delivered. The exclusion now applies to services that are neither publicly financed nor publicly mandated (lines 238-240).

11.2 Screening rule

Responding to the reviewer's main comment that it was unclear how we would operationalise this concept when selecting studies, we have added an explicit four-condition screening rule (lines 243-251). A study is eligible where the services concerned are the responsibility of a publicly accountable sub-national body; that body is responsible for a defined resident population or territory rather than only for enrolled or paying users; the services are predominantly publicly financed, irrespective of delivery arrangement; and the study reports on staff performing needs assessment, eligibility determination, formal decision-making on entitlement or service coordination and allocation. The rule will be piloted alongside the eligibility criteria and inter-rater agreement reported.

Examining the criteria against the reviewer's point about operationalisation, a further inconsistency was found: demographic trends were listed among the forms of reconfiguration, when they are a driver rather than a form of it. The section now lists organisational change, reform, new models of service delivery, decentralisation or centralisation of responsibility, and the digitalisation of assessment and decision-making as the forms of reconfiguration considered, with demographic, workforce and fiscal pressures identified as affecting the changes. We have also removed "development initiatives", which had no operational meaning for screening purposes.

***11.3** Typographical corrections*

Two typographical errors have been corrected: "To cast a wide net" (line 258) and a duplication of "language capabilities" (lines 262-263).

12. Data analysis and presentation (pages 8-9, lines 320-335)

12.1 What softwares?

Thank you for this comment, the section now specifies the tools: data extraction using a structured form developed in Excel, and coding of extracted findings in NVivo (lines 332-333). Screening software is specified under Study selection, where Rayyan was already named.

12.2 [Main comment; which descriptive data will be extracted]

This was a really valuable comment. Revising this section brought a further inconsistency with the Introduction, which states that the charting form will record the governance context of included settings, whereas the charting list recorded only country of origin. Since the review now considers services organised at municipal, communal, district, county and comparable sub-national levels, country of origin does not capture the governance arrangement that distinguishes them.

We have followed the reviewer's suggestion on extraction directly. The charting form now records context and jurisdiction, the organisational model of the responsible body, how the case manager role is defined, the population served, tasks and responsibilities, the conditions under which reconfiguration was implemented, and reported outcomes, alongside source characteristics setting, reconfiguration type and study methods (page 9, lines 323-328). We have also removed the sentence stating that reported design features would be identified and mapped, which was imprecise and duplicated the charting of study methodology.

We have also added a brief justification for combining descriptive synthesis with reflexive thematic analysis (page 9, lines 328-335). Charting provides the descriptive map required by JBI methodology. The second sub-question, however, concerns perception and sense-making, and the relevant findings will be reported in the analytic language of each included study. Reflexive thematic analysis is used to pattern these findings across differing terminologies and service systems, making the interpretive work explicit rather than implicit.

13. Ethics and dissemination (page 9, lines 338-362)

13.1 Be mindful of repetitions

Thank you for pointing this out. The section previously stated the commitment to honesty, transparency and documentation across three sentences that made substantially the same point. This is now covered in one sentence (lines 340-342), anchored to PRISMA-ScR so that the commitment is specific rather than general.

13.2 Re ethics and number of analysts

The protocol did not state how many reviewers would undertake the review, and the sections on study selection and data extraction were inconsistent, specifying "two independent reviewers" and "two or more" respectively. The section now states the number of reviewers, specifies that screening, full-text assessment and data extraction will each be carried out independently by two reviewers, and states that inter-rater agreement will be reported for the pilot (lines 343-346). The wording in the Study selection and Data extraction has been made consistent with this.

13.3 Consider using this place to clarify analyses, outcomes, contributions, applications, limitations

Thank you for this suggestion. Addressing the first of the two suggested additional sections, we have added a paragraph on outcomes, contributions and applications (lines 347-358). It describes what the review will produce – a map of the types of reconfiguration examined, of how case managers and other caseworkers experience and respond to such change, and of how their roles and practices are reported to alter – and identifies the audiences it is intended to serve: researchers, managers and policymakers in territorially responsible services, case managers and caseworkers themselves, and the doctoral project of which the review forms the first stage. Charting the governance context of included settings allows the map to be read across differing national arrangements.

Regarding the second suggested section, on potential limitations, we have expanded the existing Strengths and limitations of this study section (page 2, lines 53-72) rather than adding a separate one, since BMJ Open requires that section and a second treatment would have

duplicated it. It now notes the restriction to sources in English and Scandinavian languages, the heterogeneity of the service systems included and how the charting framework addresses it, and that scoping reviews do not include formal appraisals of methodological quality. Ethics and dissemination refer to it rather than repeating the material.

The clarification on analyses requested here is addressed in response 12.

Changes made for consistency

The revisions described above required corresponding changes to the abstract and to the Strengths and limitations section, which we set out here rather than repeating under each point.

The abstract (pages 1-2, lines 13–52) twice characterised reconfiguration as organisational change affected by demographic trends. Following our revision of the Introduction, which identifies demographic, workforce and organisational pressures rather than demographic change alone, this framing was no longer accurate and has been replaced with the forms of reconfiguration the review will consider (lines 24–27). Two sentences duplicated elsewhere in the paragraph have been removed. The abstract now defines public municipal healthcare in the opening lines as publicly financed services holding territory-based responsibility for the health of a defined resident population, at municipal or comparable sub-national level (page 1, lines 15-18), so that the concept is visible in its broadened form at first contact rather than only in the full text. The abstract now also states that no geographic limits will be applied (page 2, lines 35–36) and that the third inclusion criterion covers public municipal healthcare and equivalent sub-national arrangements (line 39). The exclusion criteria have been brought into line with the revised eligibility criteria, which now turn on direct attention to the work, roles or experiences of case managers and caseworkers rather than on the presence of substantive data (lines 39-41). The description of analysis now states that extracted data will be charted and synthesised thematically, matching the Data analysis section (line 35). The sentence on honesty and transparency has been corrected and aligned with the revised Ethics and dissemination section (lines 49-51).

The Strengths and limitations section (page 2, lines 53–72) has been revised accordingly. We have combined the two bullets describing procedural rigour (lines 54–56), which allowed us to add a point not previously made: that eligibility now rests on territory-based responsibility for a defined resident population rather than on administrative labels, and that governance context will be charted so that variation between systems is presented as a finding rather than as unexplained heterogeneity (lines 61–64). The limitation concerning language now names the languages at issue — French, Portuguese and Japanese — since these include systems we identify as relevant comparators (lines 69–71), and we have added the standard caveat that methodological quality of included sources will not be formally appraised (lines 71–72).

We are very grateful to the reviewer for the detail and care of these comments. Taken together they identified that the protocol stated what the review would do without sufficiently establishing why it was needed, where it stands, or how its central concept would be applied, and we consider that the manuscript is considerably stronger for having addressed that.
